# CTDP1 regulates breast cancer survival and DNA repair through BRCT-specific interactions with FANCI

**DOI:** 10.1038/s41420-019-0185-3

**Published:** 2019-06-19

**Authors:** Wen-Feng Hu, Kimiko L. Krieger, Dragana Lagundžin, Xueli Li, Ronald S. Cheung, Toshiyasu Taniguchi, Keith R. Johnson, Tadayoshi Bessho, Alvaro N. A. Monteiro, Nicholas T. Woods

**Affiliations:** 1 Eppley Institute for Research in Cancer and Allied Diseases, Fred & Pamela Buffett Cancer Center, University of Nebraska Medical Center, Omaha, NE 68198 USA; 2 Center for Molecular Medicine, Xiangya Hospital, Central South University, Changsha, China; 3 Mass Spectrometry and Proteomics Core Facility, University of Nebraska Medical Center, Omaha, NE 68198 USA; 4 Department of Cancer Epidemiology, H. Lee Moffitt Cancer Center & Research Institute, Tampa, FL 33612 USA; 5 Divisions of Human Biology and Public Health Sciences, Fred Hutchinson Cancer Research Center, Seattle, WA 98109 USA; 6 Department of Molecular Life Science, Tokai University School of Medicine, Isehara, Kanagawa Japan

**Keywords:** Breast cancer, Protein-protein interaction networks

## Abstract

BRCA1 C-terminal domains are found in a specialized group of 23 proteins that function in the DNA damage response to protect genomic integrity. C-terminal domain phosphatase 1 (CTDP1) is the only phosphatase with a BRCA1 C-terminal domain in the human proteome, yet direct participation in the DNA damage response has not been reported. Examination of the CTDP1 BRCA1 C-terminal domain-specific protein interaction network revealed 103 high confidence interactions enriched in DNA damage response proteins, including FANCA and FANCI that are central to the Fanconi anemia DNA repair pathway necessary for the resolution of DNA interstrand crosslink damage. CTDP1 expression promotes DNA damage-induced FANCA and FANCD2 foci formation and enhances homologous recombination repair efficiency. CTDP1 was found to regulate multiple aspects of FANCI activity, including chromatin localization, interaction with γ-H2AX, and SQ motif phosphorylations. Knockdown of CTDP1 increases MCF-10A sensitivity to DNA interstrand crosslinks and double-strand breaks, but not ultraviolet radiation. In addition, CTDP1 knockdown impairs in vitro and in vivo growth of breast cancer cell lines. These results elucidate the molecular functions of CTDP1 in Fanconi anemia interstrand crosslink repair and identify this protein as a potential target for breast cancer therapy.

## Introduction

BRCA1 C-terminal (BRCT) domains are restricted to a specialized group of only 23 proteins that are predominantly associated with DNA damage response (DDR) pathways^1–3^. The BRCT domain serves as a scaffold for organizing multi-protein complexes that orchestrate the decision between homologous recombination (HR) and non-homologous end joining (NHEJ) through the recruitment of specific repair proteins to sites of DNA damage^4–7^. In the human proteome, CTDP1 is the only phosphatase that also encodes a BRCT domain^1^, suggesting it could play a unique role by regulating the phosphorylation-mediated signaling involved in the DDR. Knockout of *CTDP1* (*Fcp1*) in yeast increases sensitivity to DNA-damaging agents without affecting DNA damage-induced Serine-2 phosphorylation in the RNA polymerase II C-terminal domain^8^, suggesting that CTDP1 could participate in the DDR in a transcription-independent manner.

Because of their role as protein scaffolds, the mechanisms by which BRCT domains perform their unique functions in the DDR can be elucidated through analysis of their protein interactions. The goal of this project was to evaluate CTDP1 BRCT domain protein interactions to identify its underlying role in the DDR, which identified DNA interstrand crosslink (ICL) repair proteins FANCA and FANCI. These repair proteins function in the Fanconi anemia (FA)-BRCA DNA repair pathway and regulate sensitivity to ICL-generating compounds^9^. In the FA-BRCA pathway, the FANCI–FANCD2 complex is activated by mono-ubiquitination via the FA core complex and phosphorylation by the DNA damage kinase ATR^10–12^. Activation of the FANCI–FANCD2 complex is responsible for the recruitment of downstream proteins necessary for the removal and repair of ICL^13,14^. Removal of FANCD2 from stalled replication forks is regulated by the deubiquitinating enzyme USP1-UAF1 and is necessary following repair to allow for replication restart^15^. The phosphatase(s) regulating FANCI and FANCD2 activation status and chromatin localization state is unknown, but the interactions we identified between FANCI and CTDP1 strongly implicated this phosphatase in the regulation of FA proteins in response to ICL.

Examining the functional link between CTDP1 and the ICL repair pathway, we have determined that CTDP1 expression enhances HR repair efficiency. We also discovered that CTDP1 expression promotes FANCA and FANCD2 foci formation and regulates FANCI chromatin localization and association with the DNA damage foci marker γ-H2AX. Participation in the DDR is further demonstrated by hypersensitivity to MMC, melphalan, 5-fluorouracil (5-FU), cisplatin, and ionizing radiation (IR) in breast cell lines with knocked down CTDP1 expression. In addition, CTDP1 expression is necessary for breast cancer cell line growth both in vitro and in vivo. This study defines DNA damage-specific functions of CTDP1 through BRCT-mediated interactions observed with FA pathway proteins and establishes CTDP1 as a regulator of breast cell sensitivity to DNA-damaging agents.

## Results

### CTDP1 BRCT domain interactions reveal interactions with FA proteins

**Fig. 1:**
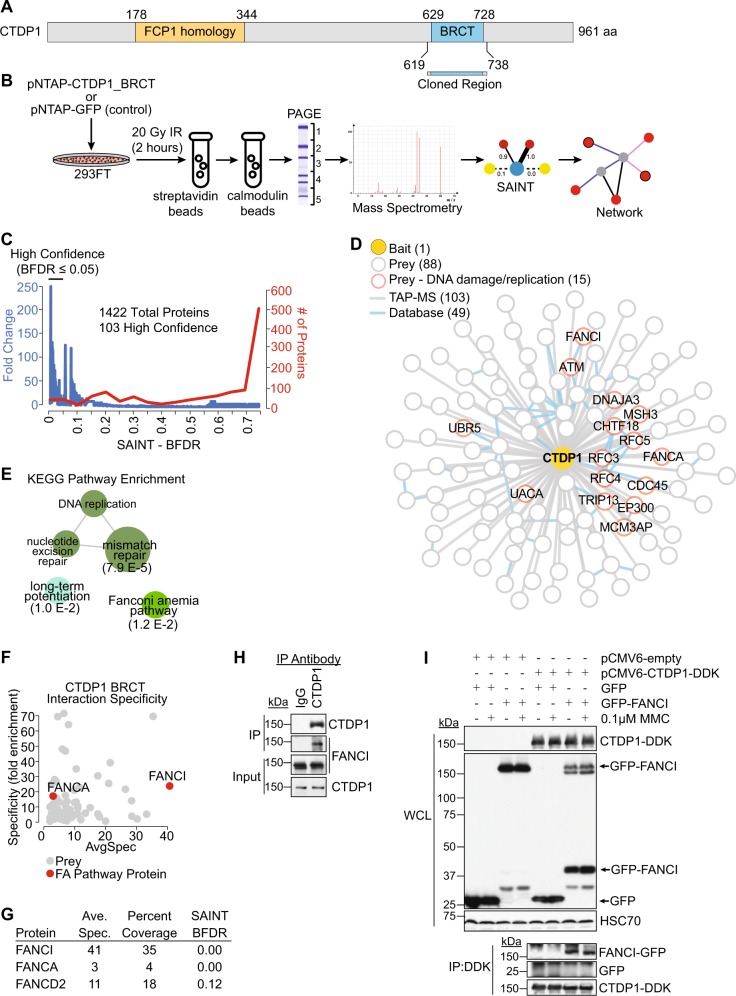
CTDP1 BRCT domain TAP-MS identifies interactions with Fanconi anemia proteins. **a** Representation of CTDP1 and its modular domain organization. The BRCT region cloned for TAP–MS experiments is indicated between amino acids 619–738. **b** Workflow diagram of the TAP–MS experiment. **c** SAINT output for Bayesian false discovery rate (SAINT-BFDR) was used to create a high confidence interaction list. Graph represents the fold change in protein representation between CTDP1 BRCT purification and control and the number of proteins at binned intervals of 0.5 SAINT-BFDR scores. **d** TAP–MS experiments generate a comprehensive interaction network of 103 proteins interacting with the CTDP1 BRCT domain. Red outlined nodes indicate proteins with GO annotations for DNA damage (GO:0006281) and DNA replication (GO:0006260). Edge color represents source of protein interactions (gray: TAP-MS, blue: BisoGenet). **e** KEGG pathway enrichment determined by ClueGO of the 103 CTDP1 interacting proteins identified by TAP–MS, excluding CTDP1 itself. The most significant term for each cluster is presented in bold font and term *p*-value corrected with Bonferroni step down is presented in parentheses. Threshold for visualization was *p*-value ≤ 0.05 and represented inversely proportional to node size. Exact *p*-values are displayed under most significant group node determined by two-sided hypergeometric test corrected using Bonferroni step down method. **f** Specificity of protein interactions with the CTDP1 BRCT domain in comparison to 27 other BRCT domain interaction datasets. FANCI and FANCA passing SAINT-BFDR cutoff of ≤0.05 are represented as red circles. **g** Table detailing central FA pathway proteins’ (FANCA, FANCI, and FANCD2) average spectrum abundance (Ave. Spec.), peptide coverage of protein sequence (Percent Coverage), and SAINT BFDR from TAP-MS. **h** Analysis of CTDP1 and FANCI expression in the input and immunoprecipation (IP) of control (IgG) or endogenous CTDP1 in complex with FANCI from untreated 293FT cells. **i** Top: Whole cell lysate (WCL) blot of GFP-FANCI and CTDP1-DDK constructs co-overexpressed in 293FT cells with and without 0.1 μM MMC treated for 24 h. Bottom: IP of DDK-tagged CTDP1 co-immunoprecipitates GFP-tagged FANCI independent of MMC treatment

The CTDP1 protein encodes both a phosphatase domain (FCP1 homology) and BRCT domain (Fig. 1a). We used a domain-centric approach to study the CTDP1 BRCT-dependent interactions (Fig. 1a). Human embryonic kidney 293FT cells were used because they are highly transfectable with excellent protein production characteristics^16^. Enrichment of the CTDP1 BRCT domain and its interacting partners was achieved through tandem affinity purification coupled to mass spectrometry (TAP–MS) (Fig. 1b)^1^. Affinity purifications were performed 2 h following 20 Gy IR to induce DDR kinases that phosphorylate BRCT domain-interacting motifs^17^. In total, 1422 proteins were identified in the CTDP1 BRCT TAP–MS experiments (Table S1). High confidence CTDP1 BRCT interactors were distinguished from low confidence and contaminating proteins using TAP-tagged green fluorescent protein (pNTAP-GFP) interaction experiments (*n* = 6) along with data deposited in the CRAPome (*n* = 282) as control dataset inputs into the Significance Analysis of Interactome (SAINT) algorithm^18,19^. The high confidence TAP interaction dataset for CTDP1 contains 103 proteins identified with a Bayesian false discovery rate (BFDR) ≤ 0.05 (Fig. 1c, d; Tables S1and S2). These 103 CTDP1 BRCT interacting proteins were incorporated into a Cytoscape^20^ network and 49 known protein–protein interactions were imputed from BisoGenet^21^ (Figs. 1d and S1). We were particularly interested in identifying CTDP1 BRCT-dependent interactions with DDR-associated proteins because of the conserved role of BRCT domains in this pathway^1–3,22^. A total of 15 proteins representing more than 14% of this interaction network were identified with gene ontology (GO) associations for DNA damage and DNA replication (Figs. 1d and S1). Prominent DNA damage proteins include ATM kinase, mismatch repair protein MSH3, and ICL repair proteins FANCA and FANCI.

An enrichment analysis was performed on the 103 high confidence interactions to evaluate guilt-by-association roles of the CTDP1 BRCT domain using ClueGO^23^. This analysis revealed mismatch repair, FA, and long-term potentiation KEGG pathway enrichments (Fig. 1e; Table S3). This analysis also identified a cluster of biological processes gene ontology terms related to nuclear DNA replication (GO:0033260) (Fig. S2A; Table S4). This was confirmed using an orthogonal analysis pipeline of BiNGO^24^ and Enrichment Map^25^, which identified two predominant clusters associated with both DNA replication/nucleotide excision repair and DNA damage repair (Fig. S2B). These findings support the predicted function of the CTDP1 BRCT domain with DNA repair processes.

FANCI and FANCA were plotted on an interaction specificity map using ProHits-viz^26^, which reveals that both interactors are specific to the CTDP1 BRCT domain compared to other BRCT domain interactomes (Fig. 1f). FANCI had the highest representation of ICL repair-associated protein spectra and percent protein coverage (Fig. 1f, g), which provided the justification for further analysis of the impact of CTDP1 on the ICL repair pathway through FANCI. Co-immunoprecipitation confirmed the protein–protein interaction between endogenous full-length CTDP1 and FANCI in 293FT cells (Fig. 1h). The interaction between exogenously expressed CTDP1 and FANCI was also examined in the presence and absence of ICL damage, but the interaction remains unaltered in response to this stimulus (Fig. 1h). Co-overexpression of CTDP1 with FANCI results in a decrease in full-length FANCI and the appearance of distinct lower molecular weight products (Fig. 1i).

### CTDP1 regulates FANCI chromatin localization and S/TQ motif phosphorylations

**Fig. 2:**
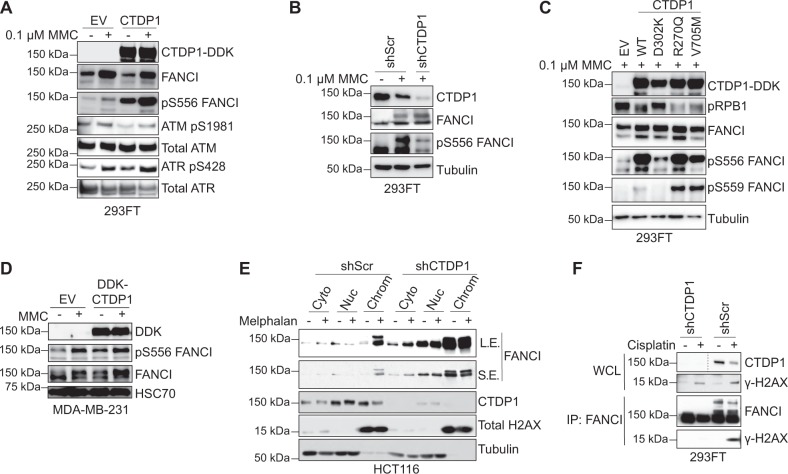
Overexpression of CTDP1 regulates FANCI SQ motif phosphorylation at S556 and S559 and chromatin localization. **a** FANCI SQ motif phosphorylation at S556 (pS556) and activation of ATM and ATR was evaluated with the indicated antibodies in 293FT cells transfected with control empty vector (EV) or CTDP1-DDK-tagged constructs in the presence and absence of DNA damage with 0.1 μM MMC for 16 h. **b** FANCI SQ motif phosphorylation at S556 (pS556) upon CTDP1 knockdown and MMC treatment. **c** Wild-type (WT), phosphatase-dead (D302K), and cancer mutants (R270Q and V705M) constructs of CTDP1 were expressed in 293FT cells treated with MMC, and the impact on SQ motif phosphorylation sites at S556 and S559 were evaluated by western blot with the indicated antibodies. **d** FANCI phosphorylation at S556 was evaluated with and without 0.4 μM MMC in MDA-MB-231 cells transfected with either EV or DDK-CTDP1 expression constructs. **e** shScr and shCTDP1 HCT116 cells were treated with or without 100 µM melphalan for 6 h and subjected to cellular fractionation and both CTDP1 and FANCI localization to chromatin was determined by western blot. Total H2AX and tubulin are included as fractionation control for chromatin and soluble protein fractions. Short exposure (S.E.). Long exposure (L.E.) **f** FANCI association with the DNA damage marker γ-H2AX was evaluated by immunoprecipitation of FANCI (IP:FANCI) followed by western blot for γ-H2AX 293FT cells either untreated or treated with 50 μM cisplatin for 16 h. Dashed line in the CTDP1 blot indicates the image has only been modified to maintain the correct order of samples

FANCI phosphorylation at amino acid S556 in the S/TQ motif is a marker of early FANCI activation prior to ubiquitination^27^. Overexpression of wild-type CTDP1 in 293FT cells promotes the robust phosphorylation of endogenous FANCI at S556 in both the absence and presence of MMC treatment (Fig. 2a). The phosphorylation of the S/TQ motif sites is dependent on activation of DNA damage kinases ATM and ATR, and we observed an interaction between CTDP1 and ATM in the TAP–MS experiments (Fig. 1d). Overexpression of CTDP1 produces an increase in the activating phosphorylation of ATR at S428, but did not affect the ATM activation-associated phosphorylation at S1981. The amount of phosphorylated S556 FANCI observed is more in the untreated CTDP1 overexpressing cells, where ATR activation is slightly lower than the treated empty vector (EV)-transfected cells (Fig. 2a). These results suggest that the induction of FANCI S556 phosphorylation could be partially dependent upon CTDP1-induced activation of ATR signaling, but other unknown mechanisms could also be affecting these FANCI phosphorylations. We confirmed that the levels of CTDP1 expression are directly related to FANCI S556 phosphorylation using shRNA-mediated knockdown of CTDP1 (Fig. 2b).

FANCI phosphorylation at both the S556 and S559 sites was elevated by the overexpression of wild-type CTDP1, but not phosphatase-dead D302K mutant (Fig. 2c). Two CTDP1 mutations (R270Q and V705M) found in TCGA and classified as “probably damaging” by Polyphen-2 were also evaluated. However, both mutations are still catalytically active toward RPB1 and promote elevated phosphorylation of FANCI at S556 comparable to wild-type CTDP1, but phosphorylation at S559 is elevated in comparison to wild-type CTDP1 (Fig. 2c). The induction of FANCI S556 phosphorylation by CTDP1 overexpression was confirmed using the triple-negative breast cancer cell line MDA-MB-231 (Fig. 2d) but was lower in signal intensity than the experiments performed in 293FT cells.

HCT116 is a colon cancer cell line routinely employed to evaluate the ubiquitination and chromatin localization of FANCI in the evaluation of the ICL repair pathway^27–30^. FANCI is ubiquitinated and localized to chromatin even in the absence of exogenous ICL damage with melphalan when CTDP1 expression is knocked down in HCT116 cells (Fig. 2e). Immunoprecipitation of FANCI in 293FT cells with knocked down expression of CTDP1 leads to a decrease in the FANCI interaction with the DNA damage foci marker γ-H2AX following treatment with cisplatin (Fig. 2f). Immunoprecipitation of FANCI also reveals a banding pattern of FANCI indicative of posttranslational modifications that are only observed with expression of CTDP1 (Fig. 2f). These results suggest CTDP1 affects multiple aspects of FANCI activation, including phosphorylation, mono-ubiquitination, and recruitment to sites of DNA damage necessary for ICL repair.

### CTDP1 promotes HR DNA repair

**Fig. 3:**
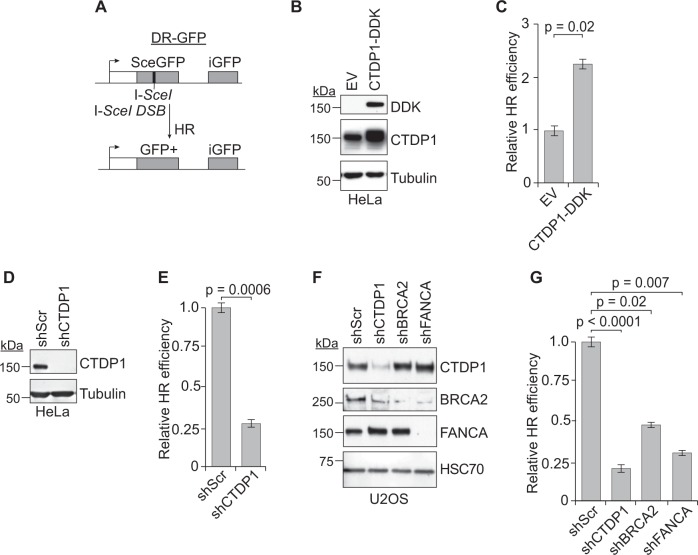
CTDP1 promotes homologous recombination DNA repair. **a** Schematic of the DR-GFP reporter homologous recombination assay. **b** Expression of transfected CTDP1-DDK in HeLa-DR cells. **c** HR efficiency of transfected cells validated in panel **b** was measured by flow cytometry quantitation of GFP-positive cells. *n* = 3. Data represented as mean ± standard error of the mean (SEM); paired two-tailed Student’s *t*-test. **d** Knockdown of CTDP1 (shCTDP1) and non-targeting scrambled shRNA control (shScr) in HeLa-DR cells evaluated by western blot. **e** HR efficiency in response to CTDP1 knockdown from cells validated in panel **d** was measured by flow cytometry quantitation of GFP-positive cells. *n* = 3; mean ± SEM; paired two-tailed Student’s *t*-test. **f** Confirmation of protein expression knock down in shScr, shCTDP1, shFANCA, and shBRCA2 targeted cells by western blot for the indicated proteins. **g** HR efficiency was measured by flow cytometry quantitation of GFP-positive cells in the cells validated in panel **f**. *n* = 3; mean ± SEM; paired two-tailed Student’s *t*-test

Proteins that participate in the ICL repair pathway, including FANCA and BRCA2, regulate HR repair efficiency that can be quantified using the DR-GFP assay (Fig. 3a)^31–33^. The impact of CTDP1 protein expression on HR efficiency was evaluated with the DR-GFP assay using established HeLa-DR and U2OS-DR cell models with chromosomal integration of the DR-GFP reporter construct. DDK epitope-tagged wild type CTDP1 (CTDP1-DDK) was overexpressed in HeLa-DR cells, leading to a more than two-fold increase in HR efficiency (Fig. 3b, c). CTDP1 knockdown using lentiviral-delivered shRNA in HeLa-DR cells produced an approximately four-fold decrease in HR efficiency (Fig. 3d, e), suggesting that HR efficiency is directly correlated with CTDP1 expression levels. We evaluated the impact of CTDP1 knockdown on HR in comparison to knockdown of either FANCA or BRCA2 proteins using the same DR-GFP reporter system in the U2OS-DR cell line (Fig. 3f). Individual knockdown of CTDP1, BRCA2, and FANCA protein expression significantly impairs HR efficiency compared to control (Fig. 3g). These experiments provide evidence that CTDP1 expression modifies HR efficiency, which could affect the response of cancers to DNA damage-inducing therapies.

### CTDP1 expression in response to melphalan does not affect ATM activation

Breast cell lines were chosen for analysis in this study on CTDP1 because a subset of BRCT domain-containing proteins are known hereditary breast and ovarian cancer susceptibility genes, such as *BRCA1* and *BRCA2*^34^. Mutations in the BRCT domain of BRCA1 increase susceptibility to breast cancer by altering its molecular function through impaired protein interactions^35^. *CTDP1* transcript expression is elevated in breast cancer samples compared to normal tissues in TCGA data queried through UALCAN^36^ (Fig. S3A). Increased *CTDP1* expression occurs in stage 1 breast cancer and maintained through stage 4 (Fig. S3B), and is elevated in both luminal and triple-negative subclasses (Fig. S3C). Breast cancer survival in the TCGA dataset was not significantly affected by *CTDP1* expression (*p* = 0.52) (Fig. S3D), but a separate database assembled by PRECOG^37^ of 16 different breast cancer studies determined that elevated *CTDP1* expression correlates with decreased survival (*z*-score = 2.2, *p* = 0.028) (Fig. S3E).

**Fig. 4:**
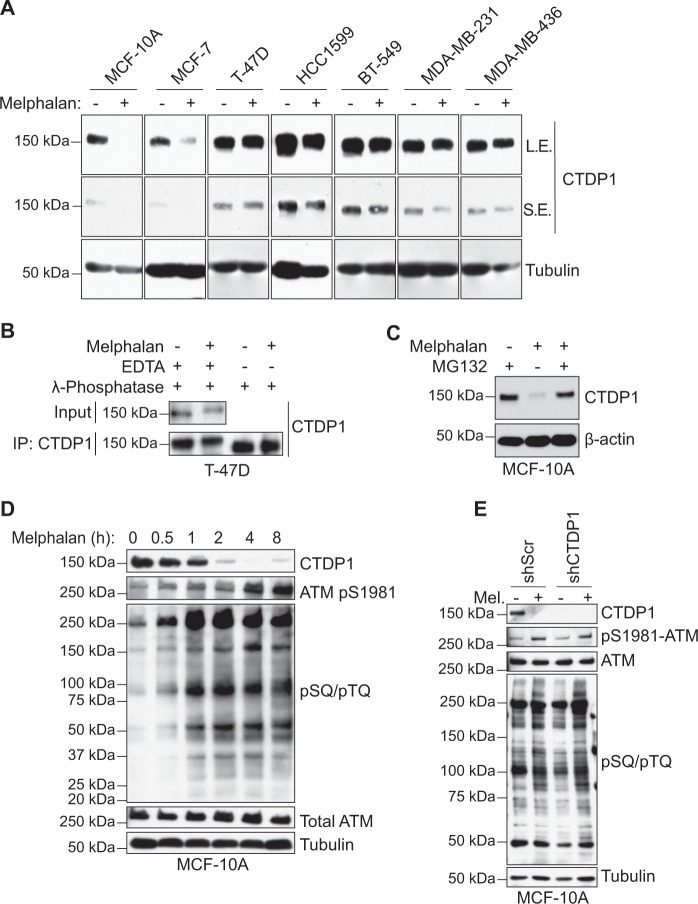
Impaired CTDP1 expression in response to DNA damage does not impact ATM activation. **a** Western blot analysis of CTDP1 expression profiles across a panel of seven breast cell lines with and without treatment of 100 µM melphalan for 6 h. **b** λ-phosphatase treatment of immunoprecipitated CTDP1 (IP: CTDP1) in T-47D cells either untreated or treated with 100 μM melphalan for 6 h. **c** MCF-10A breast cells were treated with or without 100 μM melphalan in the presence or absence of 50 μM proteasome inhibitor MG132 pretreatment for 6 h. Cell lysates were analyzed by western blot for CTDP1 and β-actin protein expression. **d** Western blot of MCF-10A lysates for CTDP1, total and phospho-ATM (pS1981), pSQ/pTQ motifs, and Tubulin collected at the indicated times following treatment with 100 μM melphalan. **e** Western blot analysis of the indicated proteins in MCF-10A cells expressing shScr control or shCTDP1 with and without treatment of 100 μM melphalan for 6 h

Regulation of protein expression and posttranslational modifications in response to DNA damage are characteristics suggestive of DDR involvement^38–40^. CTDP1 expression across a panel of seven breast cell lines revealed that 5/6 cancer lines maintained their CTDP1 expression following ICL damage with melphalan (Fig. 4a). Normal breast-derived MCF-10A and ER-positive MCF-7 breast cancer cells exhibit a reduction of CTDP1 expression in response to melphalan (Fig. 4a). A small decrease in the electrophoretic mobility of CTDP1 was observed by western blot when the cells were treated with melphalan (Fig. 4a), which was abrogated by λ-phosphatase treatment (Fig. 4b), revealing that CTDP1 is actively phosphorylated following the induction of DNA damage signaling. Since CTDP1 phosphorylation is observed in all cell lines treated with melphalan, this modification may not correlate with CTDP1 stability. CTDP1 has phosphorylation sites at S/TQ motifs (S338 and T340) curated on PhosphoSitePlus (www.phosphosite.org) that could be targeted by ATM or ATR. However, treatment with ATM or ATR inhibitors did not prevent the phosphorylation or degradation of CTDP1 observed in control cells (Fig. S4). The reduction in CTDP1 expression in MCF-10A cells caused by melphalan treatment is rescued by proteasomal inhibitor MG132 treatment (Fig. 4c).

Because of the interaction we observed between the CTDP1 BRCT and ATM (Fig. 1d), we sought to evaluate whether loss CTDP1 expression is associated with ATM activation in response to melphalan treatment in breast cells. Expression of CTDP1 is diminished after only 2 h of melphalan treatment, which corresponds with the induction of DNA damage signaling through ATM activation (Fig. 4d). However, ATM activation in response to melphalan treatment was found to be independent of CTDP1 expression in MCF-10A cells (Fig. 4e), which is in concordance with our previous observations in 293FT cells using an overexpression system (Fig. 2a).

### CTDP1 expression promotes ICL-induced FANCA and FANCD2 foci formation

**Fig. 5:**
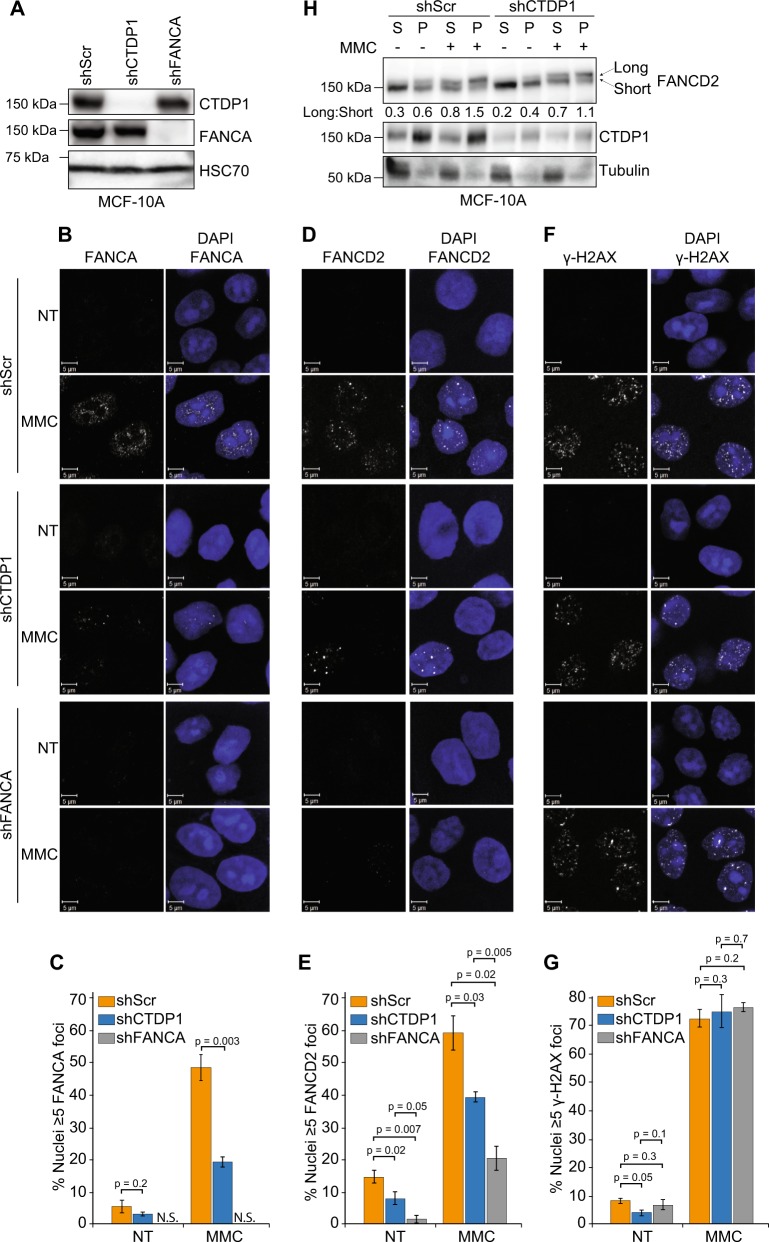
CTDP1 knockdown impairs FANCA and FANCD2 DNA damage-induced foci. **a** Western blot demonstrating knockdown of CTDP1 and FANCA using targeted shRNAs in MCF-10A cells. **b** Representative immunofluorescence of FANCA alone and merged with DAPI (white and blue color, respectively) in MCF-10A cells expressing the indicated shRNAs (shScr, shCTDP1, or shFANCA) treated with 0.2 μM MMC for 24 h. Scale = 5 μm. **c** Graph representing the percentage of nuclei containing ≥ 5 FANCA foci with or without MMC treatment. *n* = 3 independent experiments; mean ± SEM; paired two-tailed Student’s *t*-test; ≥200 cells evaluated/experiment. **d** Representative immunofluorescence of FANCD2 alone and merged with DAPI (white and blue color, respectively) in MCF-10A cells expressing the indicated shRNAs (shScr, shCTDP1, or shFANCA) treated with 0.2 μM MMC for 24 h. Scale = 5 μm. **e** Graph representing the percentage of nuclei containing ≥ 5 FANCD2 foci with or without MMC treatment. *n* = 3 independent experiments; mean ± SEM; paired two-tailed Student’s *t*-test; ≥ 100 cells evaluated/experiment. **f** Representative immunofluorescence of γ-H2AX alone and merged with DAPI (white and blue color, respectively) in MCF-10A cells expressing the indicated shRNAs (shScr, shCTDP1, or shFANCA) treated with 0.2 μM MMC for 24 h. Scale = 5 μm. **g** Graph representing the percentage of nuclei containing ≥ 5 γ-H2AX foci with or without MMC treatment. *n* = 3 independent experiments; mean ± SEM; paired two-tailed Student’s *t*-test; ≥ 200 cells evaluated/experiment. **h** Analysis of FANCD2 mono-ubiquitination using cellular fractionation of MCF-10A cells (shScr and shCTDP1) with and without MMC treatment for 24 h by calculating the ratio of long (mono-ubiquitinated): short (non-ubiquitinated) proteoforms quantified by LI-COR signal intensities. (S, soluble protein fraction, including cytosol and nucleoplasm; P, pellet protein fraction, contains chromatin)

Activated FANCA and FANCD2 localize to sites of ICL damage and are visible as distinct nuclear foci by immunofluorescence^14,41^. To evaluate the impact of CTDP1 on FANCD2 foci formation, we knocked down either CTDP1 or FANCA, as a control, in MCF-10A cells (Fig. 5a). The specificity of the FANCD2 antibody used to perform immunofluorescence was validated by western blot (Fig. S5A) and immunofluorescence (Fig. S5B) in FANCD2-null PD20 cells with and without reconstitution of exogenous FANCD2 expression. The FANCA antibody was validated by immunofluorescence, which did not detect FANCA signal in the shFANCA cells (Fig. 5b). Knockdown of CTDP1 significantly impairs FANCA foci in response to MMC treatment (Fig. 5b, c). CTDP1 knockdown also results in decreased FANCD2 foci formation compared to control, but this was to a lesser extent when compared to the control FANCA knockdown (Fig. 5d, e). Upstream γ-H2AX foci formation was not impacted by CTDP1 or FANCA knockdown (Fig. 5f, g). CTDP1 knockdown only marginally affects FANCD2 mono-ubiquitination (Fig. 5h). CTDP1 cellular localization was also examined in T-47D cells by immunofluorescence, and while some punctate CTDP1 foci were found that localized to both the cytoplasm and the nucleus, their prevalence is not affected by DNA damage and nuclear co-localization with FANCD2 foci is observed only in a few instances (Fig. S6).

### CTDP1 knockdown increases sensitivity to DNA damage

**Fig. 6:**
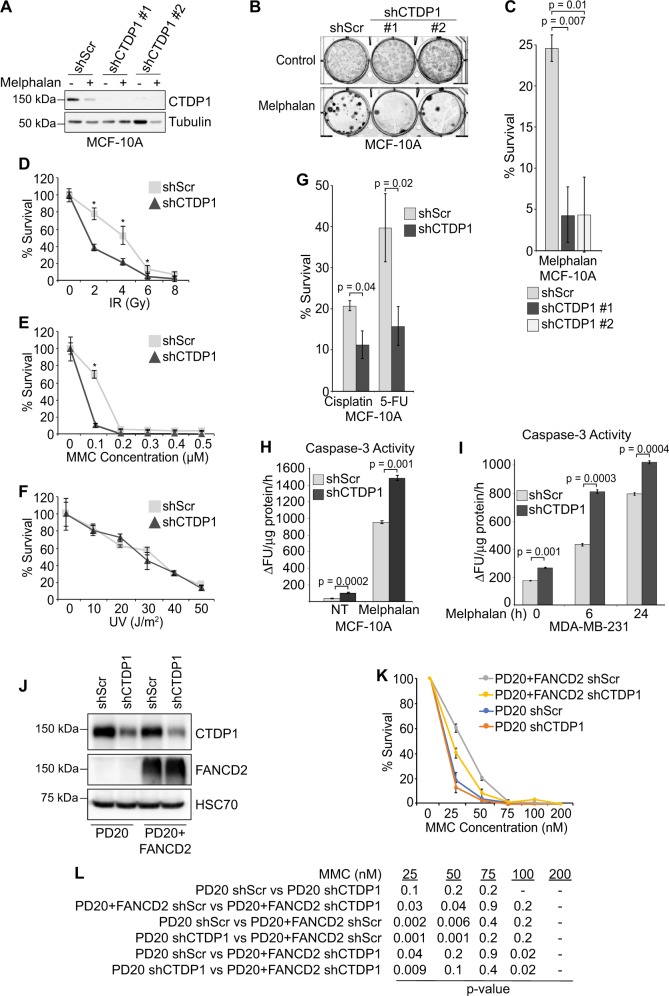
CTDP1 knockdown promotes ICL sensitivity and ICL-induced apoptosis. **a** Western blot confirmation of shRNA targeting CTDP1 with two independent shCTDP1 constructs (#1-TRCN0000002996 and #2-TRCN0000436164). **b** Representative clonogenic survival assay in MCF-10A cells expressing non-targeting shScr and shCTDP1 constructs treated with control or 5 μM melphalan. **c** Quantification of the clonogenic survival assays using MCF-10A cells expressing shScr or shCTDP1 #1 treated with 5 μM melphalan (Mel). *n* = 3 independent experiments. Data represented as mean ± SEM; paired two-tailed Student’s *t*-test. **d** Clonogenic survival assay using MCF-10A cells expressing shScr or shCTDP1 #1 treated with 0, 2, 4, 6, or 8 Gy ionizing radiation. *n* = 3; mean ± SEM; Asterisks indicate *p*-value ≤ 0.05; paired two-tailed Student’s *t*-test. **e** Clonogenic survival assay using MCF-10A cells expressing shScr or shCTDP1 #1 treated with 0, 0.1, 0.2, 0.3, 0.4, or 0.5 μM MMC. *n* = 3; mean ± SEM; Asterisks indicate *p*-value ≤ 0.05; paired two-tailed Student’s *t*-test. **f** Clonogenic survival assay using MCF-10A cells expressing shScr or shCTDP1 #1 treated with 0, 10, 20, 30, 40, or 50 J/m^2^ ultraviolet radiation. *n* = 3; mean ± SEM. **g** Clonogenic survival assay using MCF-10A cells expressing shScr or shCTDP1 #1 treated with 5 μM cisplatin or 12 μM 5-Fluorouracil (5-FU). *n* = 3; mean ± SEM; paired two-tailed Student’s *t*-test. **h** Caspase-3 activity assay using MCF-10A cells expressing shScr or shCTDP1 treated with control or melphalan for 24 h. *Y*-axis units are represented as the change in fluorescent units per microgram protein extract per hour of reaction incubation (ΔFU/μg protein/h). *n* = 3; mean ± SEM; paired two-tailed Student’s *t*-test. **i** Caspase-3 activity assay using MDA-MB-231 cells shScr or shCTDP1 treated with control or Melphalan for 0, 6, and 24 h. *n* = 3; mean ± SEM; paired two-tailed Student’s *t*-test. **j** Western blot confirmation of CTDP1 knockdown and FANCD2 expression in PD20 (FANCD2 deficient) and PD20 + FANCD2 fibroblasts. **k** Clonogenic survival assay using PD20 and PD20 + FANCD2 cells expressing shScr or shCTDP1 #1 treated with 0, 25, 50, 75, 100, or 200 nM MMC. *n* = 3; mean ± SEM. **l** Table of *p*-values determined using the paired two-tailed Student’s *t*-test for each listed comparison of treatment groups from results in panel **k**

Cells with a defective DDR are more sensitive to DNA damaging agents^42,43^. We performed clonogenic survival assays in MCF-10A cells and found that targeted knockdown of CTDP1 using two unique shRNAs (Fig. 6a) decreases the number of surviving colonies in comparison to control cells treated with melphalan (Fig. 6b, c). Similar effects were also observed for other DNA-damaging treatments, including IR to generate double-strand breaks and mitomycin c to induce ICL, in which CTDP1 knockdown decreases the survival of these cells following treatment (Fig. 6d, e). However, survival in response to cyclobutane–pyrimidine dimers caused by ultraviolet radiation is not affected by CTDP1 knockdown (Fig. 6f). In addition, survival in response to cisplatin, generating intra-strand DNA crosslinks and ICL, or 5-FU, that causes double-strand breaks and replication inhibition, is decreased following CTDP1 knockdown (Fig. 6g).

To determine if the decreased survival to ICL damage observed upon CTDP1 knockdown is attributable to an increase in cell death, caspase-3 activity was evaluated and found to be significantly elevated when CTDP1 is knocked down in both MCF-10A and MDA-MB-231 cell lines treated with melphalan in comparison to shScr control (Fig. 6h, i). We also examined the sensitivity to ICL caused by knockdown of CTDP1 in the context of FANCD2 deficiency using human fibroblast cells derived from a FA patient which lacks a functional FANCD2 gene (PD20) and the FANCD2-reconstituted cell line (PD20+FANCD2)^44^. (Fig. 6j). Knockdown of CTDP1 in FA pathway-proficient, PD20+FANCD2 cells led to a decrease in cell survival, but this was intermediate to the sensitivity caused by loss of FANCD2 expression in the PD20 shScr cells (Fig. 6k, l). Knockdown of CTDP1 in FANCD2-deficient PD20 cells did not lead to a further decrease in cell survival compared to PD20 shScr control (Fig. 6k, l), suggesting an intact FA repair pathway is necessary to observe an increase in cell sensitivity to ICL caused by knockdown of CTDP1.

### CTDP1 knockdown impairs breast cancer cell growth in vitro and in vivo

**Fig. 7:**
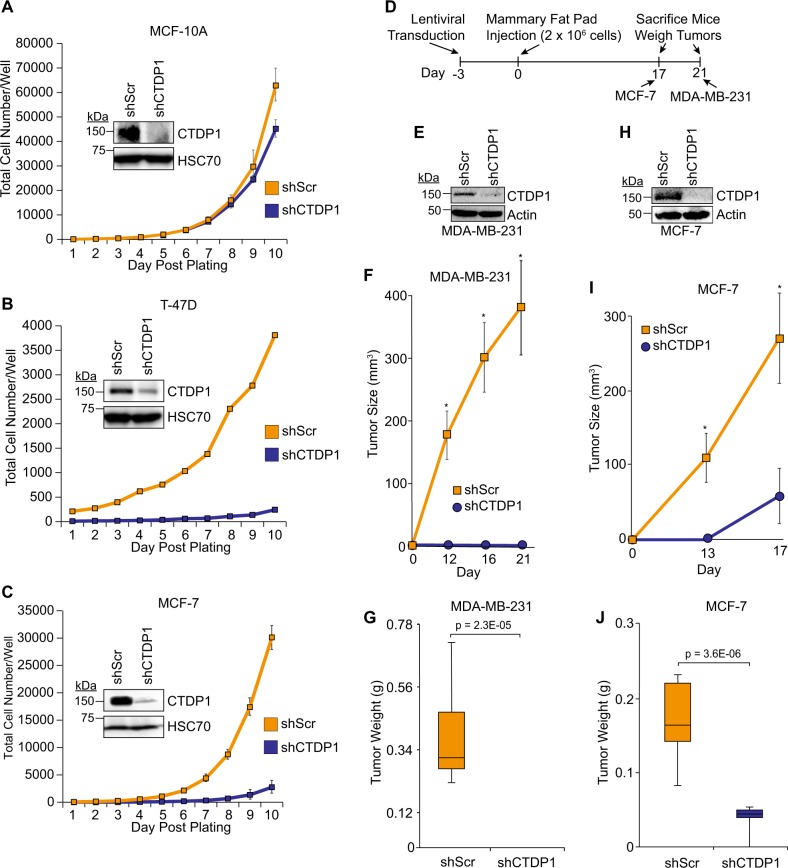
CTDP1 knockdown inhibits breast cancer cell line growth in vitro and in vivo. In vitro growth curves of breast cell lines (**a**) MCF-10A, (**b**) T-47D, and (**c**) MCF-7 comparing cell proliferation between shScr and shCTDP1 expressing cells. *n* = 3; mean ± SEM. **d** Timeline and experimental design of the in vivo xenograft mouse experiments. **e** Western blot confirming CTDP1 knockdown in MDA-MB-231 cells used for this xenograft study. **f** Volumes of shScr (*n* = 10) and shCTDP1 (*n* = 12) MDA-MB-231 tumors grown in mice at Day 0, 12, 16, and 21 using Vernier caliper measurements of palpable tumors. Mean ± SEM; Asterisks indicate *p*-value ≤ 0.05; two-tailed Student’s *t*-test. **g** Weights of shScr and shCTDP1 MDA-MB-231 tumors obtained at necropsy. Box spans the interquartile range, horizontal line represents median, ±highest and lowest observations. **h** Western blot confirming CTDP1 knockdown in MCF-7 cells used for this xenograft study. **i** Volumes of shScr (*n* = 10) and shCTDP1 (*n* = 6) MCF-7 tumors grown in mice at Day 0, 13, and 17 using Vernier caliper measurements of palpable tumors. Mean ± SEM; Asterisks indicate *p*-value ≤ 0.05; two-tailed Student’s *t*-test. **j** Weights of shScr and shCTDP1 MCF-7 tumors obtained at necropsy. Box spans the interquartile range, horizontal line represents median, ±highest and lowest observations

To further validate CTDP1 as a potential target to sensitize breast cancer cells to chemotherapy, we sought to evaluate the impact of targeted deletion or depletion of CTDP1 expression on breast cell line growth. Repeated attempts to knockout *CTDP1* by CRISPR in MDA-MB-231 and MCF-7 cells failed to yield viable cultures. Information available from Depmap.org indicates that CTDP1 is a “common essential” gene because CRISPR-mediated knockout of this gene significantly reduced cell viability in 505 out of 517 cancer cell lines tested (Fig. S7). Therefore, we have had to employ incomplete knockdown techniques using shRNA to maintain viable cell cultures. We have determined that knockdown of CTDP1 is well-tolerated by the MCF-10A cell line with a minor reduction in cell growth in vitro (Fig. 7a). However, T-47D and MCF-7 breast cancer cell lines display reduced growth rates when CTDP1 is knocked down (Fig. 7b, c).

Breast cancer can be classified according to distinct molecular subtypes, and the most aggressive subtype is triple-negative (ER^−^, PR^−^, HER2^−^)^45^. We chose the MDA-MB-231 and MCF-7 cell lines to interrogate two unique subtypes of breast cancer in vivo. MDA-MB-231 is a triple-negative breast cancer cell line. Both the MCF-7 and T-47D breast cancer cell lines interrogated above in the in vitro proliferation analysis are classified as Luminal A (ER^+^, PR^+/−^, HER2^−^), and we chose to utilize MCF-7 to represent this subtype for the in vivo analysis. The MDA-MB-231 and MCF-7 cancer cell lines were subjected to CTDP1 knockdown and then injected into the mammary fat pad of immunodeficient mice to evaluate in vivo growth (Fig. 7d). MDA-MB-231 shScr cells formed tumors, but shCTDP1 cells did not (Fig. 7f), and at necropsy shCTDP1 tumors were unidentifiable (Fig. 7g; Fig. S8A). MCF-7 cells were also subjected to CTDP1 knockdown for this xenograft study (Fig. 7h). Palpable tumors were observed from the MCF-7 shScr cells at day 13 but were not observed in the shCTDP1 group until day 17 and only 5/6 injections resulted in tumors (Fig. 7i). The shCTDP1 tumors were also found to be significantly smaller by weight at necropsy (Fig. 7j; Fig. S8B). CTDP1 depletion in vitro and in vivo diminishes tumorigenic potential of breast cancer models, which is also consistent with previous findings in gastric and lung cancer in vitro experiments^46,47^. These data suggest that CTDP1 could be a promising target for cancer therapeutic development.

## Discussion

Here we provide evidence that CTDP1 participates in the DDR through protein–protein interactions with ICL repair proteins FANCA, FANCI, and FANCD2. CTDP1 expression levels directly correlate with cellular HR efficiency, and knockdown of CTDP1 promotes sensitivity to ICL DNA damage. CTDP1 knockdown also prevents the formation of both FANCA and FANCD2 foci, which are essential for ICL repair. CTDP1 expression regulates FANCI S/TQ motif phosphorylation, chromatin localization, and interaction with γ-H2AX. Together, these results suggest that CTDP1 plays an important role in the regulation of the ICL repair pathway and reveal that it may be a promising therapeutic target for breast cancer.

Mutations in the *CTDP1* gene have not been identified in FA patients. However, mutation of human *CTDP1* is associated with the rare congenital cataracts facial dysmorphism neuropathy (CCFDN) syndrome^48–51^. Homozygous mutation of an *Alu* element in intron 6 (IVS6+389C>T) of *CTDP1* leads to the generation of ~70% non-functional truncated protein and 30% functional full-length protein^48^. CCFDN-affected individuals exhibit a number of phenotypic abnormalities and some features are shared with FA patients including short stature, sub-normal weight, and hypogonadism^52^. However, there is no documented association of CCFDN with cancer incidence. Complete functional loss of CTDP1 may be embryonic lethal in humans, as previously observed in *D. melanogaster*^53^. Currently, no mammalian genetic models of targeted *CTDP1* deletion exist, but will be important to decipher the in vivo role of CTDP1 in the regulation of FA proteins and ICL repair.

CTDP1 expression promotes FANCI SQ motif phosphorylations at S556 in a phosphatase-dependent manner. There are several possible explanations for this seemingly contradictory observation. For instance, CTDP1 could dephosphorylate FANCI outside of the SQ motif sites interrogated in this study to regulate the substrate accessibility or phosphorylation stability of SQ motif residues. Another potential explanation for the increased SQ motif phosphorylations on FANCI caused by CTDP1 overexpression could be the impact on ATM or ATR kinase activation. Overexpression of CTDP1 promotes increased pS428 ATR, but this does not appear to fully explain the level of FANCI phosphorylation caused by CTDP1 overexpression. Previous studies in *D. melanogaster* found that either CTDP1 overexpression or knockdown promotes cell death in a manner independent of ATM or ATR function^54^, but the role of FANCI in this cell death process requires further analysis.

In conclusion, CTDP1 represents a unique BRCT domain containing protein with functional associations with the ICL repair pathway. Understanding the role phosphatases play in the DDR could expand strategies to modify cancer sensitivity to DNA damage-based therapies.

## Materials and methods

### Cell culture and transfection

The breast cell lines and HCT116 cells were purchased from ATCC. 293FT cells were purchased from Invitrogen. MCF-10A cells were cultured in DMEM/F12 medium (Invitrogen), containing 5% horse serum (Invitrogen, No. 16050-122), 20 ng/mL EGF (Sigma), 0.5 μg/mL hydrocortisone (Sigma), 100 ng/mL cholera toxin (Sigma), 10 μg/mL insulin (Sigma), and 1% penicillin–streptomycin (Invitrogen) at 37 °C in 5% humidified CO_2_ incubators. HCC1143, HCC1599, and MDA-MB-231 cells were cultured in RPMI medium (Invitrogen), containing 10% FBS and 1% penicillin-streptomycin. BT-549 cells were cultured in RPMI medium containing 10% FBS, 1% penicillin–streptomycin and 0.023 IU/mL insulin. MDA-MB-436 cells were cultured in DMEM medium containing 10% FBS, 1% penicillin–streptomycin, and 10% NEAA. T-47D cells were cultured in RPMI medium containing 10% FBS, 1% penicillin–streptomycin, and 0.2 U/mL insulin. MCF-7 and 293FT cells were cultured in DMEM medium (Invitrogen), containing 10% FBS and 1% penicillin–streptomycin. HCT116 cells were cultured in McCoy’s 5A medium (Invitrogen), containing 10% FBS, 1% penicillin–streptomycin. PD20 and PD20 cells with reconstituted FANCD2 expression were a gift from the laboratory of Dr. Alan D’Andrea. Transfection of 293FT cells to generate lentiviral particles was performed using the calcium phosphate method and the ViraPower system (Invitrogen). Target cells were transduced with virus, and 48 h later, selected on 1 μg/mL puromycin for 7–10 days.

### Plasmids, primers, and shRNAs

The BRCT domain of CTDP1 with 10 amino acid additional sequence flanking the domain regions (amino acids 619–738) was cloned into a pNTAP construct using InterPlay N-Terminal Mammalian TAP Vectors (Agilent Technologies) using the EcoRI and HindIII restriction sites. Primers: CTDP1-forward 5′-TAGAATTCGCGCCGGACATCCGCAAGATCG-3′ and CTDP1-reverse 5′-CTAAGCTTTCACTCCCTCTGTGCCTTGGTGTGA-3′. Full-length FANCI transcript variant 1 C-terminal fusion with monomeric GFP (mGFP) construct in pLenti-C-mGFP-P2A-Puro was purchased from Origene. Full-length CTDP1 transcript variant 1 C-terminal fusion with myc and DDK epitopes in pCMV6-Entry was purchased from Origene. CTDP1 mutant plasmids were designed and made using the QuikChange II XL Site-Directed Mutagenesis Kit (Agilent). The associated primers were generated by using the online QuikChange Primer Design program (Agilent). Primers: CTDP1-R270Q-forward 5′-CGAGAAGAAGCTTTTTTCTCACCAAATATTATCAAGGGATGAATGTA-3′ and CTDP1-R270Q-reverse 5′-TACATTCATCCCTTHATAATATTTGGTGAGAAAAAAGCTTCTTCTCG-3′; CTDP1-D30K-forward 5′-GTGGAGACTCAATGGTTTGCATTATTAAGGATCGAGAAGATGTCTG-3′ and CTDP1-D302K-reverse 5′-CAGACATCTTCTCGATCCTTAATAATGCAAACCATTGAGTCTCCAC-3′; CTDP1-V705M-forward 5′-GCGGACACCTGCACATGGTCAACCCTGAC-3′ and CTDP1-V705M-reverse 5′-GTCAGGGTTGACCATGTGCAGGTGTCCGC-3′. The shRNAs were purchased from Sigma Aldrich and were provided by The RNA Consortium shRNA Library. shRNAs: BRCA2 (TRCN0000009825), CTDP1 (TRCN0000002996) and (TRCN0000436164), FANCA (TRCN0000296799), and non-targeting shRNA control (shScr) (SHC016).

### Mass spectrometry

The InterPlay TAP Purification Kit (Agilent Technologies) and NETN buffer was used to purify pNTAP-CTDP1 and its interactors as previously described^1^. Protein fractions (*n* = 4) created by SDS–PAGE were excised, destained, reduced with tris–carboxyethylphosphine, alkylated with iodoacetamide, and digested overnight with sequencing-grade trypsin (Promega). Tryptic peptides were eluted from the gel and concentrated to 20 μL by vacuum centrifugation. A nanoflow liquid chromatograph (U3000, Dionex) coupled to an electrospray ion trap mass spectrometer (LTQ-Orbitrap, Thermo) was used for tandem mass spectrometry (MS/MS) peptide sequencing experiments. Samples were first loaded onto a pre-column (5 mm Å–300 mm inside diameter (ID), C18 PepMap100, Dionex) and washed for 8 min with aqueous 2% acetonitrile and 0.04% trifluoroacetic acid. With a flow rate of 300 nL/min, the trapped peptides were eluted onto the analytical column (75 mm ID Å~15 cm, C18 PepMap 100, Dionex). The 60-min gradient program began at 95% solvent A (aqueous 2% acetonitrile/0.1% formic acid) for 8 min; solvent B (aqueous 90% acetonitrile/0.1% formic acid) was ramped from 5% to 50% over 35 min. Then, solvent B was ramped from 50% to 90% B over 5 min, followed by washing and re-equilibration of the column. The mass spectrometer cycled through a survey scan, and five tandem mass spectra were collected in a data-dependent manner in the linear ion trap using 60 s exclusion for previously sampled peptide peaks. The CTDP1 BRCT domain raw mass spectrometry data files have been deposited to the ProteomeXchange Consortium via the PRIDE^55^ partner repository with the project accession: PXD009541 and project 10.6019/PXD009541.

### Mass spectrometry data analysis

Database searches were conducted against human entries in the SwissProt database (v.20130501) using Mascot (Matrix Science, version 2.2.04)^56^, assuming the digestion enzyme trypsin was used and allowing as many as two missed cleavages. Tandem mass spectra were matched to peptide sequences with a peptide ion mass tolerance of 1.2 Da and a fragment ion mass tolerance of 0.80 Da. Oxidation of methionine and carbamidomethylation of cysteine were specified as variable modifications. Assignments were manually verified by inspection of the tandem mass spectra and coalesced into scaffold reports (v4.8.4, available at http://www.proteomesoftware.com) for statistical analysis and data presentation.

Scaffold was used to validate MS/MS-based peptide and protein identifications. Peptide identifications were accepted if they could be established at >95.0% probability as specified by the PeptideProphet algorithm^57^. Protein identifications were accepted if they could be established at >50.0% probability and contained at least two identified peptides. Protein probabilities were assigned by the ProteinProphet algorithm^58^. Proteins that contained similar peptides and could not be differentiated based on MS/MS analysis alone were grouped to satisfy the principles of parsimony.

Significance Analysis of INTeractome (SAINT, v2.4.0)^59^ was used to score CTDP1 BRCT domain interaction specificity from mass spectrometry results by comparing proteins identified in the CTDP1 interaction screen to six negative control pNTAP-GFP performed in-house and another 282 control interaction datasets downloaded from the CRAPome (crapome.org)^18^. The top 10 highest peptide counts for each protein from the control datasets were used to evaluate interaction specificity. Interactions obtained from the CORUM database were used to boost scores of known protein complexes in the sample^60^. The SAINT output was also manually inspected to remove known calmodulin-binding proteins and carryover contaminants. For visual and analysis purposes, ProHits-viz was used to evaluate the output from SAINT^26^.

### Network visualization and gene ontology analysis

The CTDP1 BRCT high confidence interaction network consisting of 103 proteins was uploaded to Cytoscape for visualization and analysis. This set of 103 interacting proteins was analyzed with ClueGO (version 2.2.5)^23^ to determine GO Biological Processes (version 09.02.2016) and KEGG Pathways (version 26.10.2017) with significant enrichments in this dataset with evidence codes of “All_without_IEA”. GO Term fusion was selected and only statistically significant enriched pathways with *p*-value ≤ 0.05 were shown. Statistical significance was determined using a two-sided hypergeometric test corrected with Bonferroni step down method. BiNGO^24^ was used for a similar analysis using the hypergeometric test and Benjamini & Hochberg false discovery (FDR) correction at a significance level of 0.05. The output of BiNGO was used as the input for Enrichment Map^25^ as a network-based method to visualize and interpret the protein-set enrichment results from BiNGO.

### HR DR-GFP reporter assay

A HeLa-derived cell line, HeLa-DR-13-9 (generous gift from Dr. Jeffrey Parvin at the Ohio State University) was used to measure HR activity. For overexpression experiments, HeLa-DR-13-9 cells were seeded at 3 × 10^4^ cells per well in a six-well plate one day before transfection. The cells were transfected with each pCMV6 expression plasmid and incubated for 2 days. For suppression experiments, HeLa-DR-13-9 cells, as well as U2OS cells with stable-expressing DR-GFP substrate, were seeded at 5 × 10^4^ cells per well in a 12-well plate one day before lentiviral infection. The cells were infected by each shRNA-containing lentivirus and incubated for one day. The infected cells were grown for an additional one day in fresh medium. For the induction of double-strand breaks and measurement of HR activity, the cells were transfected by the expression plasmid of I-*SceI* endonuclease (generous gift from Dr. Maria Jasin at Memorial Sloan Kettering Cancer Center). HR activity was determined by FACS 3 days after transfection.

### Western blotting and antibodies

Whole cell lysates were prepared using NETN lysis buffer (10 mM HEPES pH 7.4, 10 mM KCl, 0.05% NP-40). The lysis buffer contained phosphatase inhibitors (50 mM NaF, 10 mM β-glycerophosphate, 0.1 mM NaVO_4_) and protease inhibitor cocktail (Sigma). Samples were mixed 1:4 with 5X Laemmli buffer and incubated at 95 °C for 5 min. Approximately 50–100 μg of protein was prepared for loading. The membrane was blocked in 5% non-fat milk and incubated with the primary antibody. BRCA2 (A300-005A), CTDP1 (A301-172A), FANCA (A301-980A), FANCI (A300-212A), and H2AX (A300-082A) antibodies were purchased from Bethyl Laboratories, Inc. ATM (#2873), Phospho-(Ser/Thr) ATM/ATR Substrate Motif (#9607), Phospho-ATR (Ser 428) (#2853), ATR (#13934), and phosphor-RPB1 CTD (Ser2) (#13499) antibodies were purchased from Cell Signaling Technology. Phospho-ATM S1981 (AF1655) antibody was purchased from R&D Systems, Inc. FANCD2 (ab108928) antibody was purchased from Abcam. Gamma-H2AX (NB100-384) antibody was purchased from Novus Biologicals. DDK (FLAG tag) (TA50011-100) and monomeric GFP(mGFP) (TA180076) antibodies were purchased from OriGene Technologies, Inc. HSC70 (sc-7298), β-actin (sc-47778), and α-tubulin (sc-53030) antibodies were purchased from Santa Cruz Biotechnology. FANCI phospho-antibodies (pS556 and pS559) were kind gifts from the Taniguchi lab^27^. Protein complexes were visualized with ECL (Thermo Scientific), or near-IR secondary antibodies (LI-COR) on the Odyssey Fc Imaging System (LI-COR).

### Immunoprecipitation

For immunoprecipitation experiments, lysates were prepared with NETN lysis buffer with protease and phosphatase inhibitors. One milligram of total protein was used for immunoprecipitation and incubated with 5 μg anti-CTDP1 or 1 μg anti-DDK antibody on ice for 1 h followed by addition of Protein A/G beads (Santa Cruz Biotechnology) and incubated at 4 °C overnight with rotation. Immunoprecipitated protein complexes on Protein A/G beads were washed a minimum of three times with NETN buffer. For phosphatase-treated samples, one unit of λ-protein phosphatase was incubated with the washed immunoprecipitated protein complexes on Protein A/G beads for 1 h at 37 °C, with agitation at 1000 rpm on a temperature-controlled orbital shaker, then washed an additional three times with NETN buffer. The immunoprecipitated complexes were boiled at 95 °C for 5 min to dissociate them from the Protein A/G beads and analyzed by western blot.

### Caspase-3 apoptosis assay

Cells were treated with 100 μM melphalan at ~50% confluence for 24 h. The cells were collected and lysed in CHAPS lysis buffer (1% CHAPS, 150 mM NaCl, 10 mM HEPES pH 7.4) with protease and phosphatase inhibitors, as previously described^61^. Approximately 25–50 μg of protein were incubated in assay buffer with Caspase-3 substrate (Sigma) in 96-well plate in dark for 1 h. The plate was read with fluorometer set at excitation 360 nm, emission 460 nm. Caspase-3 activity was assayed as DEVDase activity with the caspase-3 fluorescence assay kit (Sigma). Caspase-3 activity is reported as the change in fluorescent units per microgram protein lysate per hour of reaction incubation (ΔFU/μg protein/h).

### Clonogenic survival assay and cell proliferation

One thousand MCF-10A shScr or shCTDP1 cells were seeded in each well of six-well plates. The following drugs and agents were used to treat cells for 48 h: mitomycin C, melphalan, cisplatin, 5-FU, paclitaxel, ultraviolet (UV) radiation, and IR. The cells recovered in fresh media for 7–10 days. The plates were stained with Giemsa for 20 min, and colonies were counted. Each genotype and drug dose was done in three independent experiments. Cell proliferation experiments were performed with the indicated cell lines by plating 250 cells/well in 12-well plates. Individual wells were trypsinized daily for 10 days and total cells/well were determined by counting using a hemocytometer. Each data point was analyzed with three independent experiments.

### Immunofluorescence

Cells were grown on coverslips and treated with 0.1 μM MMC or control and fixed in 4% paraformaldehyde for 15 min. Coverslips were washed in 0.5% Triton X-100/PBS for 1 min. Coverslips were then incubated with antibodies directed toward FANCA (Bethyl, A301-980A, 1:200 dilution), FANCD2 (Abcam ab108928, 1:200 dilution), or γ-H2AX (Novus Biologicals NB100-78356, 1:200 dilution) in 3% BSA for 1 h. After coverslips were washed three times with PBS, donkey anti-rabbit AlexaFluor-647 (Abcam ab150075, dilution 1:500) or goat anti-mouse AlexaFlour-488 (Abcam ab150113, 1:500 dilution) secondary antibody was added for 1 h. Coverslips were finally washed three times with PBS and mounted with DAPI counterstain mounting solution onto microscope slides. Images were captured on a Zeiss 710 Confocal Laser Scanning Microscope using a ×63 oil objective.

### Chromatin extraction

The protocol for the acid extraction of chromatin proteins was adapted from the Mostoslavsky lab protocol and performed as previously described^62,63^. Cell pellets were suspended with lysis buffer (10 mM HEPES pH 7.4, 10 mM KCl, 0.05% NP-40) three times the pellet volume and incubated on ice for 20 min. The lysates were then centrifuged at 14,000 rpm for 10 min at 4 °C. The supernatant was collected (cytoplasmic proteins) and the pellet was washed again and centrifuged. The remaining pellet was suspended in Low Salt Buffer (10 mM Tris–HCl pH 7.4, 0.2 mM MgCl_2_, 1% Triton-X 100) three times the pellet volume and incubated on ice for 15 min. The lysates were centrifuged again at 14,000 rpm for 10 min at 4 °C. The supernatant was collected (nucleoplasmic proteins) and the pellet was suspended in three pellet volumes of 0.2 N HCl and incubated on ice for 20 min. After centrifugation at 14,000 rpm for 10 min at 4 °C, the supernatant was collected and neutralized with an equal volume of 1 M Tris–HCl pH 8 (chromatin-bound proteins).

### CSK fractionation

The protocol outlined and used was described previously^64^. Cells were lysed with CSK buffer (10 mM PIPES pH 6.8, 100 mM NaCl, 300 mM sucrose, 1.5 mM MgCl_2_, 1 mM EGTA, 1 mM EDTA, 1 mM PMSF, 50 mM NaF, 0.1 mM NaVO_4_, 0.1% Triton X-100, and Protease Inhibitor Cocktail (Sigma Aldrich) (1:100 dilution)) and incubated on ice for 5 min. After the lysate was centrifuged at 1500×*g* for 5 min, the supernatant was labeled “S.” The pellet was then washed with the same buffer, centrifuged at 1500×*g* for 5 min, and labeled “P.”

### Orthotopic xenograft tumor model

NOD scid gamma (NSG) mice were purchased from Jackson Laboratory. MDA-MB-231 or MCF-7 breast cancer cells were transduced with shScr or shCTDP1 lentiviral constructs and selected on 1.0 μg/mL puromycin for 7 days. Four-week-old female mice were injected with 2 × 10^6^ MDA-MB-231 shScr (*n* = 5 mice) or shCTDP1 (*n* = 6 mice) cells in 100 mL of 50:50 Matrigel/Collagen I into each of the left and right fourth abdominal fat pads by a small surgery at the base of the nipple. For MCF-7 xenografts, shScr (*n* = 5 mice) or shCTDP1 (*n* = 3 mice) were used. MDA-MB-231 tumor growth was monitored externally using Vernier calipers at 12, 16, and 21 days and animals sacrificed on day 21. MCF-7 tumor growth was monitored externally using Vernier calipers at day 13 and 17 and animals were sacrificed on day 17. Tumor weights were determined at the time of necropsy. The experimental protocol for this animal procedure was reviewed and approved by the Institutional Animal Care and Use Committee (IACUC) of Xiangya Hospital of Central South University.

### TCGA data

UALCAN (http://ualcan.path.uab.edu/cgi-bin/ualcan-res.pl) was used to query The Cancer Genome Atlas (TCGA) data for breast cancer and corresponding normal tissues for CTDP1 mRNA transcript expression levels according to tumor stage and subclassification as Luminal, HER2, or Triple Negative. Graphs were exported from UALCAN and modified for visual clarity.

## Supplementary information

Supplemental Material

Table S1

Table S2

Table S3

Table S4

Figure S1

Figure S2

Figure S3

Figure S4

Figure S5

Figure S6

Figure S7

Figure S8
