# Supplementary material for: CTDP1 regulates breast cancer survival and DNA repair through BRCT-specific interactions with FANCI
Source: Cell Death Discov. 2019 Jun 19;5:105. doi: 10.1038/s41420-019-0185-3 (PMC6584691; doi:10.1038/s41420-019-0185-3)
Supplement: Supplementary file 1 — Supplemental Material [file 41420_2019_185_MOESM2_ESM.docx]

**SUPPLEMENTARY INFORMATION**

**SUPPLEMENTARY TABLE AND FIGURE LEGENDS**

**Table S1. SAINT Output File Identifies High Confidence Interactions, Related to Figure 1C-D.**

This table provides output file obtained by running the SAINT algorithm on the CTDP1 BRCT domain interaction dataset (columns A-P), the additional filtering steps to remove non-specific interactions (columns Q-S), and whether the protein was considered high confidence and included in the final dataset for analysis (column T).

**Table S2.** **PSI-MITAB Format of CTDP1 BRCT Domain interactions, Related to Figure 1D.**

This table is provided to conform to the information reporting standards developed by the HUPO Proteomics Standards Initiative (PSI) to facilitate data comparison, exchange, and verification.

**Table S3:** **ClueGO Output of Enriched KEGG Pathways, Related to Figure 1E.**

This data table provides the corresponding individual and group term p-values of enriched KEGG Pathways identified using ClueGO in Cytoscape. Also provided are the proteins identified by TAP-MS with CTDP1 BRCT domain that are associated with each of the listed KEGG pathways enriched in this analysis.

**Table S4: ClueGO Output of Enriched Biological Processes, Related to Figure S2A.**

This data table provides the corresponding individual and group term p-values of enriched biological processes identified using ClueGO in Cytoscape. Also provided are the proteins identified by TAP-MS with CTDP1 BRCT domain that are associated with each of the listed GO_BP terms enriched in this analysis.

**Figure S1. Annotated CTDP1 BRCT Domain Interaction Network, Related to Figure 1D.**

This figure is the same as that represented in **Figure 1D**, but it is enlarged and expanded so that it can display the protein identity of each of the 103 high-confidence protein interactions identified by TAP-MS.

**Figure S2. Functional Annotation of CTDP1 BRCT Interacting Proteins, Related to Figure 1E.**

**A**. Gene ontology enrichment of biological processes determined by ClueGO of the 103 CTDP1 interacting proteins identified by TAP-MS, excluding CTDP1 itself. The most significant term for each cluster is presented in bold font and term P-value corrected with Bonferroni step down is presented in parentheses. Threshold for visualization was P-value ≤ 0.05 and represented inversely proportional to node size. Exact p-values are displayed under most significant group node determined by two-sided hypergeometric test corrected using Bonferroni step down method. **B.** The results from BiNGO were used as input into the Enrichment Map application in Cytoscape. Significant terms (P-value ≤ 0.05) were visualized and clusters of related terms are shown as connected clusters of nodes.

**Figure S3. CTDP1 Expression in Clinical Samples Annotated in TCGA, Related to Figure 4.**

**A**. UALCAN (http://ualcan.path.uab.edu/cgi-bin/ualcan-res.pl) was used to query TCGA level 3 RNA-seq data for breast cancer and corresponding normal tissues for CTDP1 mRNA transcript expression levels. **B**. Comparison of CTDP1 transcript expression by disease stage (Stages 1-4) of breast cancer (BRCA). **C**. Comparison of CTDP1 transcript expression by disease subclasses (Luminal, HER2, and Triple Negative) of breast cancer. **D**. Kaplan-Meier survival plot of breast cancer patients from TCGA data stratified into 2 groups corresponding to CTDP1 mRNA expression: 1) High Expression (n = 272), and 2) Low/Medium Expression (n = 809). **E**. PRECOG analysis of CTDP1 mRNA expression from studies on breast cancer. Positive z-scores are associated with decreased survival when CTDP1 expression is elevated, while negative z-scores are associated with increased survival.

**Figure S4. Evaluation of ATM and ATR inhibition on CTDP1 phosphorylation and stability in response to melphalan.** MCF-10A cells were treated with 100 μM melphalan for 6 hours and the indicated inhibitors; ATMi (KU55933), 25 µM; ATRi (AZD6738), 5 µM.

­

**Figure S5. Validation of FANCD2 Antibody Used for Immunofluorescence, Related to Figure 5.**

**A.** Western blot demonstrating the specificity of the FANCD2 antibody using FANCD2-deficient PD20 fibroblast cells with and without stable expression of exogenous FANCD2. **B.** Representative immunofluorescence of FANCD2 and DAPI (red and blue, respectively) in PD20 and PD20 cells with FANCD2 reconstituted, exogenous expression in untreated cells.

**Figure S6. Cellular Localization of CTDP1 Determined by Immunofluorescence, Related to Figure 5.**

T-47D cells were untreated or treated with 100 μM melphalan for either 2 or 6 hours to induce FANCD2 DNA damage foci observed by immunofluorescence (green). CTDP1 expression was also evaluated by immunofluorescence (red). DAPI was used as a nuclear counterstain. Images were captured on an inverted Zeiss microscope using a 100X oil immersion objective. Scale = 5 μm.

**Figure S7. CTDP1 is a Common Essential Gene Necessary for Cancer Cell Viability,**

**Related to Figure 7.**

Dependency map (www.depmap.org) graph depicting the dependency score of *CTDP1* across 517 cancer cell lines that have been tested using CRISPR knockout of CTDP1. *CTDP1* was determined to be a “common essential” gene due to the lethality of 505 cancer cell lines out of all 517 tested.

**Figure S8. CTDP1 Knockdown Prevents Breast Cancer Growth *In Vivo*, Related to Figure 7.**

**A**. Tumors obtained from shScr or shCTDP1 MDA-MB-231 injected mice sacrificed at the end of the study on Day 21. Related to Figure 7G. **B**. Tumors obtained from shScr or shCTDP1 MCF-7 injected mice sacrificed at the end of the study on Day 17. Related to Figure 7J.
