# Supplementary material for: CTDP1 regulates breast cancer survival and DNA repair through BRCT-specific interactions with FANCI
Source: Cell Death Discov. 2019 Jun 19;5:105. doi: 10.1038/s41420-019-0185-3 (PMC6584691; doi:10.1038/s41420-019-0185-3)

Figure S1. Annotated CTDP1 BRCT Domain Interaction Network, Related to Figure 1D.

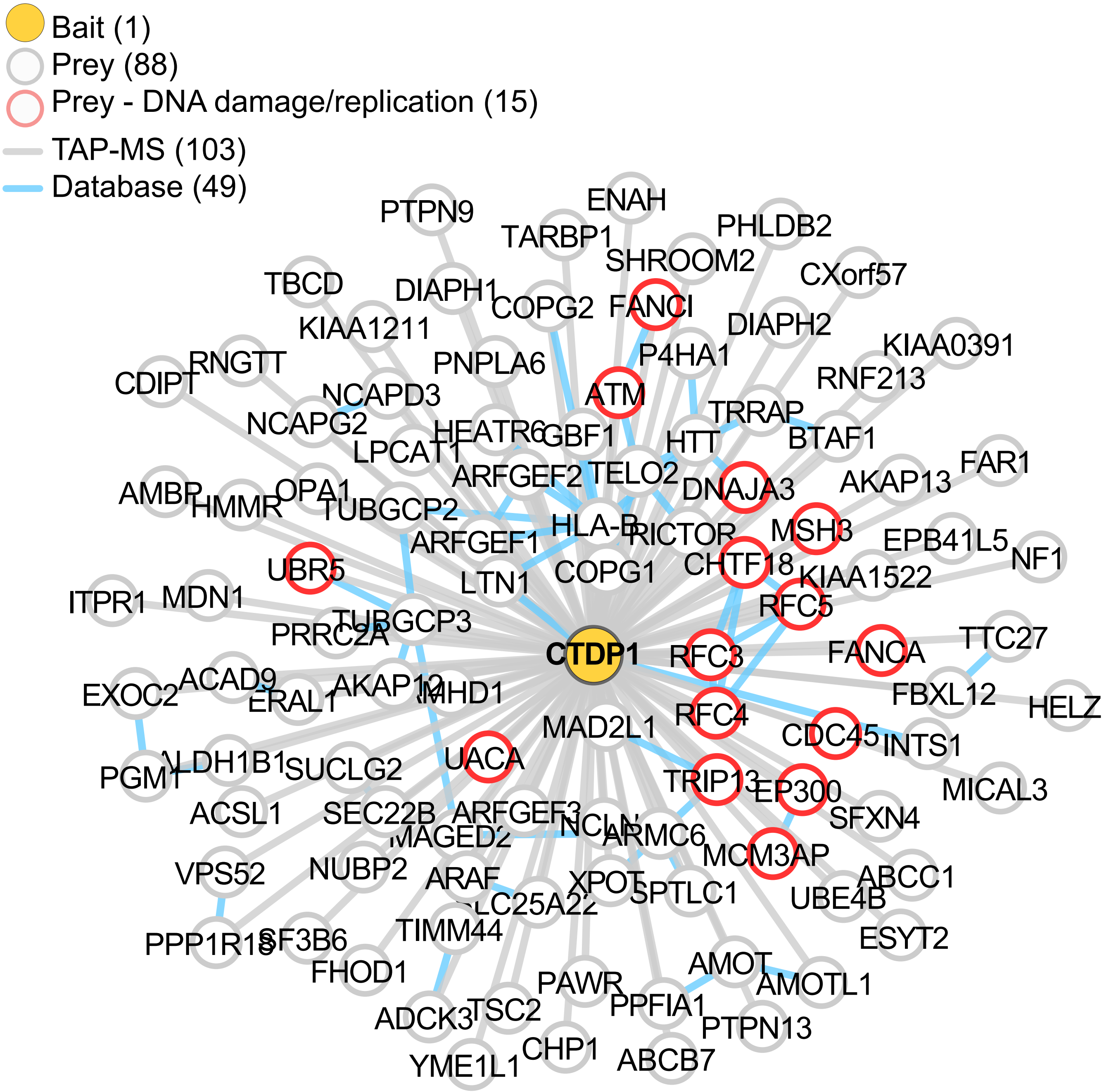

Supplement: Supplementary file 6 — Figure S1 [file 41420_2019_185_MOESM7_ESM.pdf]
