# Supplementary figures and images for: CTDP1 regulates breast cancer survival and DNA repair through BRCT-specific interactions with FANCI
Source: Cell Death Discov. 2019 Jun 19;5:105. doi: 10.1038/s41420-019-0185-3 (PMC6584691; doi:10.1038/s41420-019-0185-3)

**Figure S3. CTD1P1 Expression in Clinical Samples Annotated in TCGA, Related to Figure 4.**

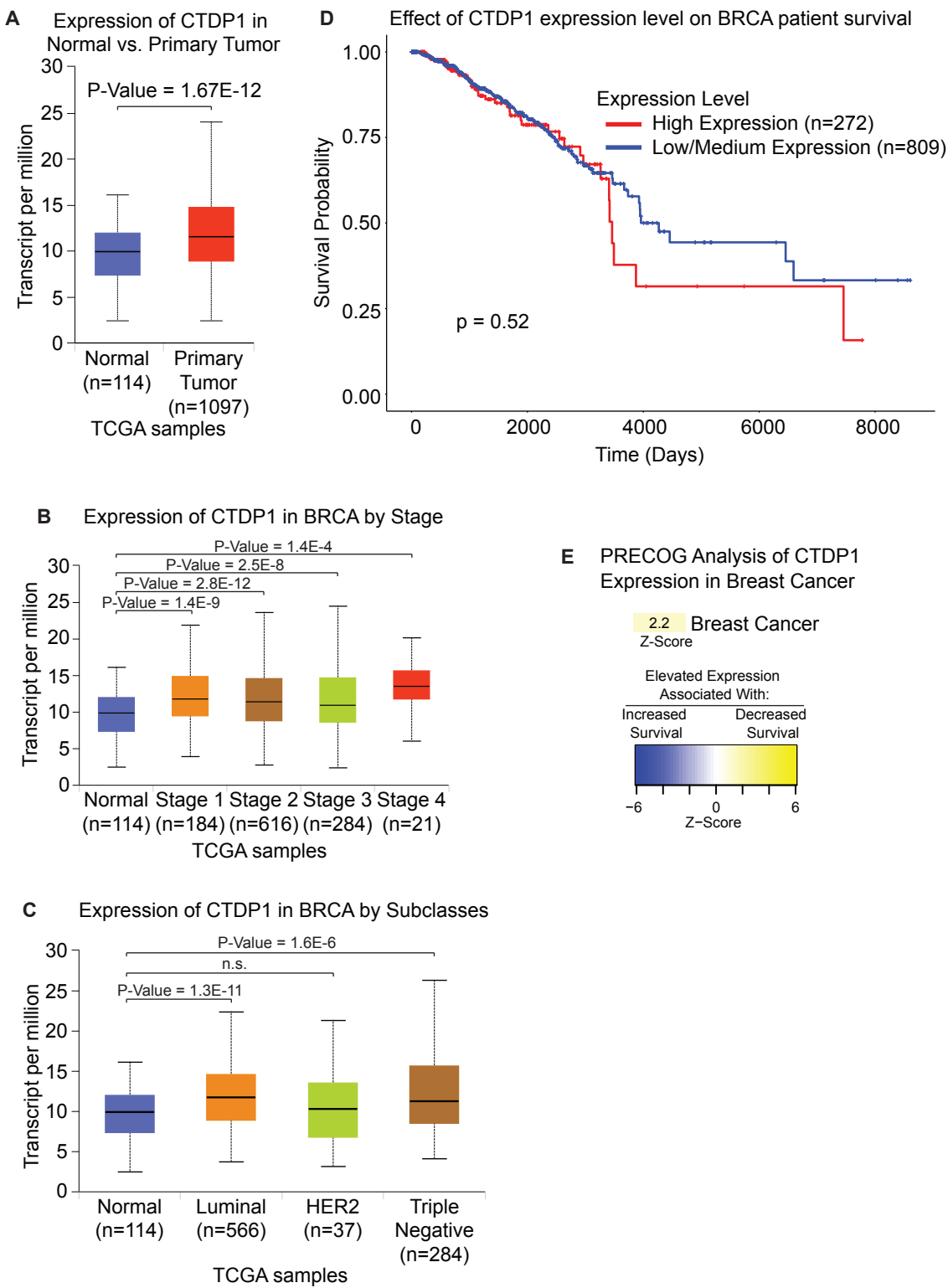

Supplement: Supplementary file 8 — Figure S3 [file 41420_2019_185_MOESM9_ESM.pdf]

**Figure S5. Validation of FANCD2 antibody used for immunofluorescence, Related to Figure 5.**

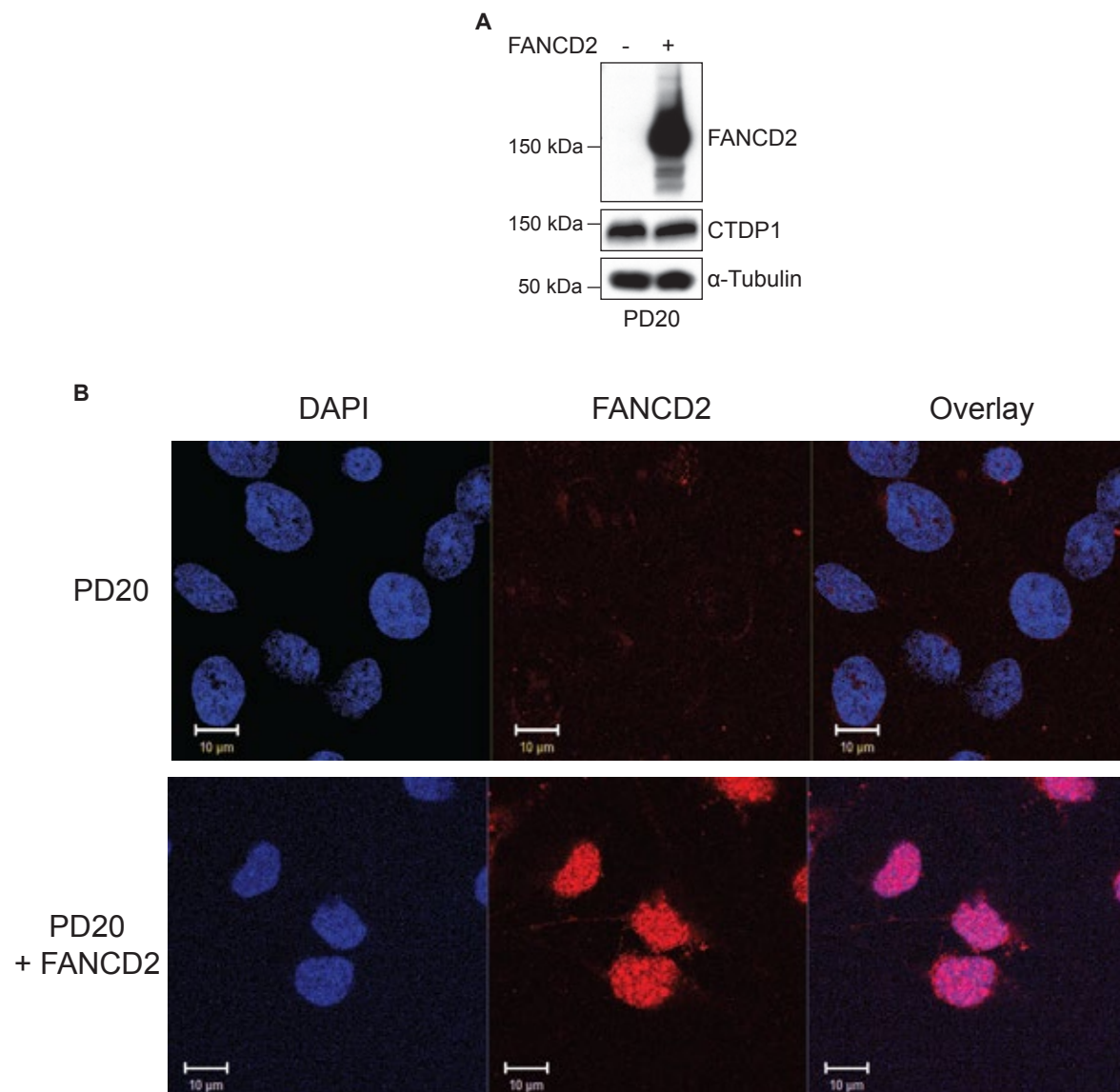

Supplement: Supplementary file 10 — Figure S5 [file 41420_2019_185_MOESM11_ESM.pdf]

Figure S6. Cellular Localization of CTDP1 Determined by Immunofluorescence, Related to Figure 5.

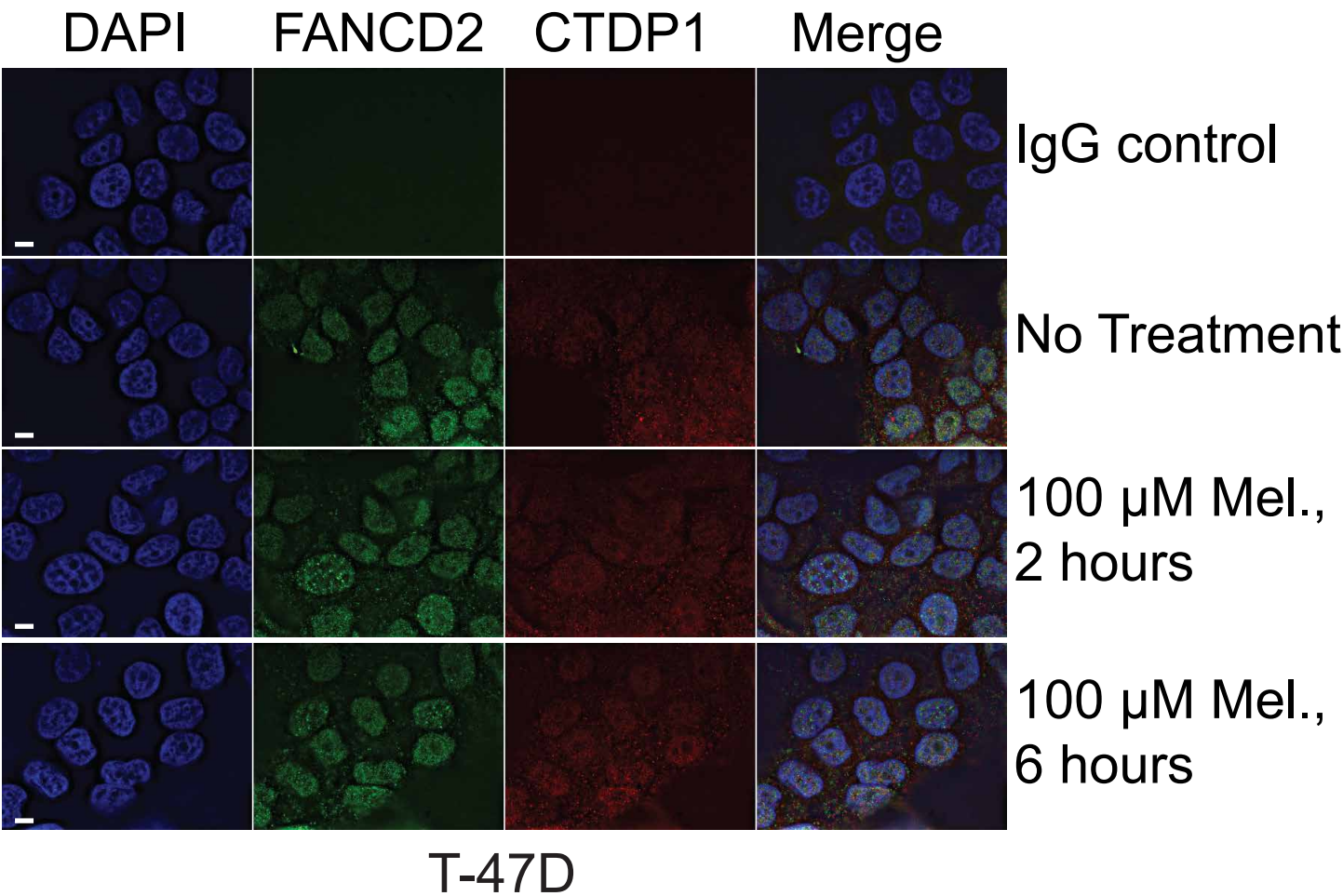

Supplement: Supplementary file 11 — Figure S6 [file 41420_2019_185_MOESM12_ESM.pdf]

**Figure S8. CTDTP1 Knockdown Prevents Breast Cancer Growth *In Vivo*, Related to Figure 7.**

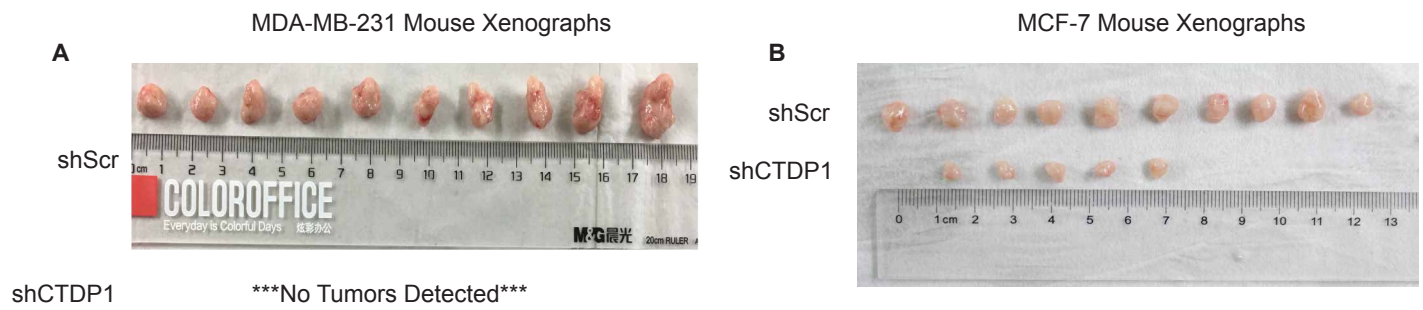

Supplement: Supplementary file 13 — Figure S8 [file 41420_2019_185_MOESM1_ESM.pdf]
